# A hospital-based study of survival in colon cancer patients of Tata Memorial Hospital (Mumbai)

**DOI:** 10.3332/ecancer.2026.2123

**Published:** 2026-05-13

**Authors:** Tushar Ambekar, Amey Oak, Sivaranjini Kannusamy, Sandhya Cheulkar, Riya R John, Shalmali Chavan, Saket Mendhe, Supriya Kadam, Mamta Lade, Ganesh Balasubramanium, Ashwin Desouza, Avanish Saklani, Rajesh Dikshit, Pankaj Chaturvedi, Sudeep Gupta

**Affiliations:** 1 Division of Cancer Care, HBCR and Survival Studies, Centre for Cancer Epidemiology (CCE); 2 Centre for Cancer Epidemiology (CCE); 3 ACTREC, Tata Memorial Centre, Homi Bhabha National Institute (HBNI), Navi Mumbai 400094, India; 4 Tata Memorial Centre, Homi Bhabha National Institute (HBNI), Mumbai 400094, India

**Keywords:** colon cancer, sociodemographic factors, clinical factors, registry, survival, India

## Abstract

**Introduction:**

Colon cancer, a type of cancer affecting the large intestine, is the fourth most common cancer worldwide, with 1,142,286 new cases and the fifth leading cause of cancer death, with 538,167 deaths reported in 2022 (GLOBOCAN). This retrospective study examines overall survival (OS) and the impact of sociodemographic and clinical factors on colon cancer patients.

**Methodology:**

The study included colon cancer patients diagnosed and treated at Tata Memorial Hospital, Mumbai, between January and December 2017, with a 5-year follow-up until 2022. Of the 761 cases registered, 434 received treatment and were eligible for analysis. OS and its variation by socio-demographics were assessed using the Kaplan-Meier method and log-rank test, while the Cox proportional hazard model variables indicate the impact of multiple factors on survival.

**Results:**

Among 434 patients (mean age 50 ± 13.6 years, 64% male), adenocarcinoma was the predominant histology (81.7%), with 98% microscopically confirmed diagnoses. Regional and distant disease were present in 32.9% and 23.5%, respectively. The 1-, 3- and 5-year OS rates were 88%, 77% and 71%. Survival was significantly higher in early-stage disease than advanced disease (5-year OS: 89% versus 43%). On multivariable analysis, advanced clinical extent (aHR 7.42; 95% CI: 3.67–14.9) and signet ring cell histology (aHR 1.95; 95% CI: 1.06–3.59) were independently associated with increased mortality, while combined-modality treatment was independently protective (aHR 0.48; 95% CI: 0.25–0.91).

**Conclusion:**

The study suggests that improving socioeconomic conditions and encouraging proactive treatment-seeking behaviour are key to increasing colon cancer survival. Cancer hospitals, together with India’s public healthcare system, including clinicians and policymakers, should implement these strategies to strengthen cancer care in low- and middle-income countries.

## Introduction

Colon cancer is an important contributor to global burden and remains a significant cause of cancer-related morbidity and mortality. According to global cancer estimates, colon cancer ranks among the leading malignancies worldwide and continues to demonstrate rising incidence in several developing regions. The increasing burden of colon cancer has been linked to urbanisation, dietary transitions characterised by high consumption of processed food and red meat, sedentary lifestyle, obesity and increasing life expectancy [1, 2]. According to GLOBOCAN 2022 estimates, colon cancer incidence ranks fourth with 1,142,286 cases and 10.7 age standardised rate (ASR), while in terms of mortality, it ranks fifth with 538,167 deaths and 4.7 ASR globally, whereas it ranks ninth with 34,046 cases in terms of incidence with 2.4 ASR, 20,629 cases while in terms of mortality, with 1.5 ASR in India [3].

In India, colon cancer incidence has shown a gradual increase in over recent decades, particularly in Urban populations. Although the incidence remains lower than that reported in Western countries, Survival outcomes remain suboptimal. Population-based cancer registry data from India have reported 5-year survival rates of colon cancer ranging between approximately 30%–40%, which is considerably lower than survival rates reported in high-income countries [4, 5]. The disparity in survival outcomes is attributable to multiple factors, including delayed diagnosis, limited awareness of early symptoms, lack of organised screening programs and unequal access to specialised oncology services [6].

Stage at diagnosis is the most important determinant of colon cancer prognosis. Early-stage colon is associated with favourable outcomes and high curative potential, primarily through surgical resection. In contrast, advanced-stage disease is associated with increased recurrence and mortality [7]. Apart from stage, several pathological characteristics influence survival outcomes. Tumour differentiation and histological subtype are recognised prognostic indicators, with aggressive variants such as signet ring cell carcinoma demonstrating poorer survival due to advanced stage at presentation and aggressive biological behaviour [8].

Treatment plays a critical role in determining survival outcomes in colon cancer. Surgical resection remains the cornerstone of curative management, while adjuvant chemotherapy has been shown to significantly improve survival in patients with high-risk disease and advanced stages. Multimodality treatment approaches have been associated with improved disease control and overall survival (OS) [9].

Despite the increasing burden of colon cancers in India, there remains limited evidence describing survival outcomes in prognostic factors specifically among colon cancer patients treated in tertiary care settings. Evaluating survival patterns and associated determinants is essential for identifying gaps in cancer care and improving treatment strategies. Therefore, the present study was undertaken to assess survival outcomes among colon cancer patients and to identify demographic, clinical, pathological and treatment related predictors of OS in a tertiary care centre, Tata Memorial Hospital (TMH), Mumbai, one of India’s cancer institutes which offers cancer diagnosis, treatment, education and research with access to a variety of cancer-related studies, treatments and patient follow-ups. TMH has a system of digital medical records that provides numerous research opportunities. This study will utilise data available in the hospital medical records on colon cancer from the year 2017 to 2022, with a follow-up period of 5 years, for the patients registered in the year 2017 at TMH, Mumbai.

## Materials and methods

This study was conducted at TMH, Mumbai. It included all colon cancer patients who were registered and received treatment at TMH between 1 January 2017, and 31 December 2017. The study was based on a review of hospital records from the TMH Cancer Registry. Data were collected using a detailed questionnaire with three sections: sociodemographic details, clinical information and follow-up. The following information was recorded: age, gender, region, occupation, education (illiterate = unable to read or write), marital status, income, tumour site, tumour type, grade, stage, type of case (new patients treated at TMH or old patients treated elsewhere before coming to TMH) and type of treatment. Colon cancer is categorised into Caecum, Appendix, Ascending colon (Right colon), Hepatic flexure, Transverse colon, Splenic flexure, Descending colon (Left colon) Sigmoid colon (Sigmoid, NOS; Sigmoid flexure; Pelvic colon), Overlapping lesions, Colon, NOS; Large intestine; Large bowel, NOS, Rectosigmoid junction, Rectosigmoid, NOS Rectosigmoid, Colon and rectum Pelvic-rectal junction. Surgery, radiation, chemotherapy or combined therapy, which is a combination of both therapies, have all been considered in the treatment analysis. Patients were followed up every 6 months. The follow-up period lasted from January 2017 to December 2022. Follow-up data were collected through the electronic medical record system and phone calls. OS was defined as the time from diagnosis to death or last follow-up. Five-year OS was calculated for patients diagnosed between January 2017 and December 2022. The study examined how different sociodemographic and clinical factors affected survival. All data were kept confidential. Data entry was done using in-house software, and analysis was performed using STATA version 15.0. Results were presented using means, standard deviations, medians, ranges, proportions and 95% confidence intervals. Survival was analysed using Kaplan–Meier and log-rank tests. The Cox proportional hazards model was used to assess the effect of multiple factors. A *p*-value of less than 0.05 was considered statistically significant. R software version 4.52 was used to generate Kaplan–Meier graphs.

## Results

The mean age was 50 ± 13.58 years; 39% (168) were aged 45–60 years and 64% (278) were male. Most patients had education up to school level (65%, 281), were employed (82%, 358), had a monthly income <INR 10,000 (53%, 229) and were married (89%, 387). Patients predominantly originated from the West (33.4%, 145) and East (33%, 142) regions. Diagnosis was confirmed microscopically in 98% (426) of cases. Adenocarcinoma was the most common histology (81.7%, 355). Tumours were well differentiated in 59% (255), while 22.4% (97) were undifferentiated. Single-modality treatment was administered to 65% (284), with 85% (367) completing treatment. New cases comprised 61% (263). At presentation, 32.9% (143) had regional disease and 23.5% (102) had distant metastases (Table 1).

**Figure 1.**
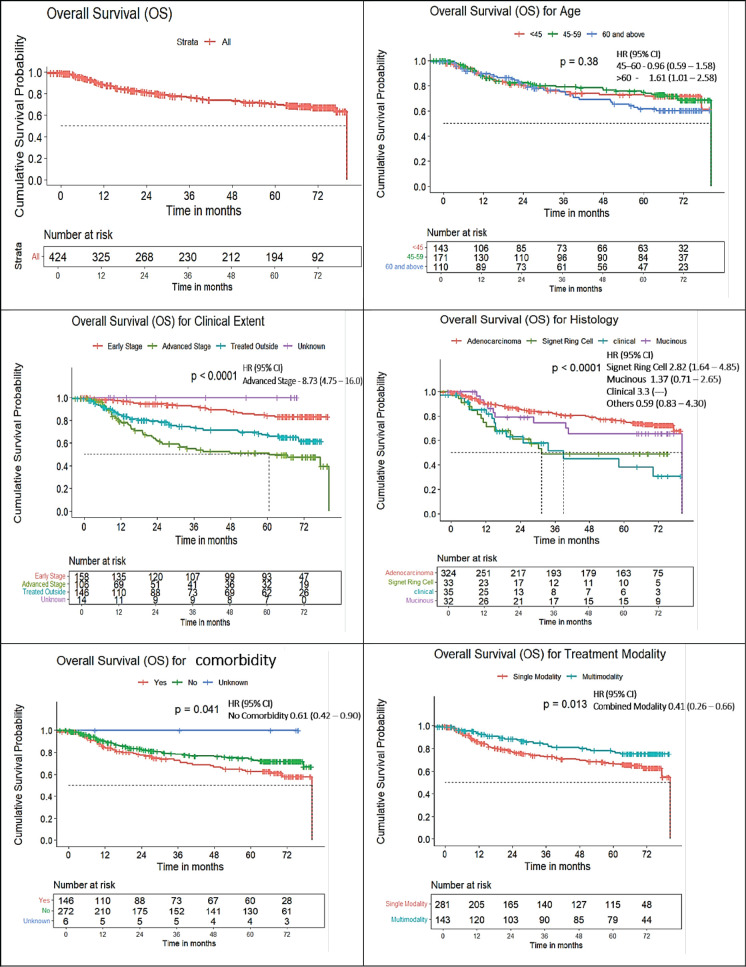
Five-year OS, survival based on the age, clinical extent, histology, comorbidity and type of treatment of colon cancer patients at TMH Mumbai, in 2017 (N = 434).

Table 2 and Figure 1 show the survival results. The overall 1-, 3- and 5-year OS rates were 88%, 77% and 71%, respectively (*n* = 434). Five-year OS by age was 75% (<44 years), 77% (45–60 years) and 58% (>60 years) (*p* = 0.04), with no significant gender difference (71% in both males and females; *p* = 0.82). Patients with early-stage disease had markedly superior survival (1-, 3-, 5-year OS: 97%, 93%, 89%) compared with those with advanced disease (73%, 50%, 43%; *p* < 0.001). By histology, adenocarcinoma showed 1-, 3- and 5-year OS of 89%, 80% and 74%, signet ring cell carcinoma 68%, 42% and 42%, and mucinous carcinoma 87%, 64% and 64% (*p* = 0.002). Survival declined with poorer differentiation (well-differentiated: 90%, 85%, 79%; poorly differentiated: 86%, 68%, 66%; undifferentiated: 81%, 53%, 51%; *p* < 0.001). Patients receiving combined-modality treatment had higher survival (93%, 85%, 81%) than those receiving single-modality treatment (84%, 71%, 64%; *p* = 0.001). Treatment completion was associated with improved outcomes (89%, 80%, 74% versus 77%, 50%, 50%;* p* < 0.001). Patients without comorbidities had better survival (89%, 79%, 75%) compared with those with comorbidities (83%, 71%, 63%; *p* = 0.02). The Kaplan–Meier graphs in Figure 1 show the graphical representation of significant variables.

On unadjusted analysis, age >60 years was associated with higher mortality (**HR 1.61; p = 0.04**), but this was not significant after adjustment. Gender and education showed no association with survival. **Signet ring cell carcinoma** remained an independent predictor of mortality (**aHR 1.95; p = 0.03**), while mucinous histology was not significant. Higher tumour grade was associated with poorer survival on unadjusted analysis only. **Advanced clinical extent** was the strongest independent predictor (**aHR 7.42; p < 0.001**). **Combined-modality treatment** was independently protective (**aHR 0.48; p < 0.001**). Treatment completion and comorbidities were not independently associated with survival after adjustment (Table 3).

## Discussion

The present study evaluated the survival outcomes among the patients diagnosed with colon cancer and identified key demographic, clinical and treatment-related predictors influencing prognosis. Our findings highlight that survival in colon cancer is strongly influenced by stage at diagnosis, treatment modalities and patient-related sociodemographic and lifestyle factor which is consistent with global and Indian literature. In our study, of the 434 patients, 24% died, while 31% were categorised as censored. The overall 1-, 3- and 5- year survival rates in our study were 88%, 77% and 71%, respectively, which appear higher than the previously reported population-based survival estimates from India. Earlier registry-based analysis have reported 5-year survival estimates for colorectal cancer ranging from 34% to 42%, reflecting poor outcomes in the general population compared to specialised tertiary centres [4 ,5]. The relatively favourable survival observed in our cohort may be attributed to improved diagnostic facilities, availability of multimodality treatment and treatment adherence in a tertiary cancer care setting.

In our study, the mean age at diagnosis was 50 years, which is lower than that reported ion western populations where colorectal cancer typically occurs after 60 years of age. Similar trends of younger age at presentation have been documented in Indian studies, which may reflect demographic differences, genetic susceptibility, lifestyle transitions and changing epidemiological patterns [10, 11]. Although older age (>60 years) was associated with increased mortality in the unadjusted hazard ratio, the association was not significant after adjustment, suggesting that treatment and stage at diagnosis may play a more critical role in determining survival than age alone. Gender did not demonstrate a significant association with survival in our study. While some international studies have reported slightly better survival among females, Indian studies have shown inconsistent findings likely due to sociocultural differences, variations in healthcare utilisation and biological heterogeneity [10, 12].

The demographic profile of our patients showed that the majority belonged to lower socioeconomic strata, with low income and educational levels. Socioeconomic status has been recognised as an important determinant of cancer outcomes, influencing health-seeking behaviour, access to diagnostic services and treatment adherence [13]. In India, disparities in healthcare infrastructure and financial constraints often contribute to delays in diagnosis and treatment, thereby adversely affecting survival outcomes [6]. Presence of comorbidities was associated with poor survival initially but was not independently predictive after adjustment. Previous studies have reported that comorbid conditions may influence treatment decisions, therapy tolerance and overall prognosis, particularly among elderly patients [14, 15].

Histological subtype significantly influenced survival outcomes in our cohort. Signet ring cell carcinoma was independently associated with increased mortality risk, whereas mucinous carcinoma was not significant after adjustment. Previous studies have demonstrated that signet ring cell carcinoma is associated with aggressive biological behaviour, advanced stage at presentation and poor response to conventional therapies, leading to inferior survival outcomes [16, 17]. Additionally, we observed declining survival with poor tumour differentiation, which is consistent with established evidence linking high-grade tumours with aggressive disease and unfavourable prognosis [18]. Stage at diagnosis emerged as the strongest independent predictor of survival in our study, with advanced-stage disease showing significantly higher mortality risk. Patients with early-stage disease demonstrated markedly superior survival compared to those with global and Indian evidence, highlighting that late-stage presentation is a major contributor to poor colorectal cancer outcomes in low- and middle-income countries [5, 19]. In India, the absence of organised population-based screening programs and limited awareness regarding early symptoms often result in diagnosis at advanced stages [20].

Treatment-related factors played a crucial role in survival outcomes. Patients receiving combined modality treatment demonstrated significantly improved survival and remained independently protective after adjustment. Multimodal therapy, including surgery combined with chemotherapy and/or radiotherapy, has been shown to improve survival by reducing recurrence and improving disease control, particularly in advanced-stage disease [7, 9]. These findings underscore the importance of multidisciplinary cancer management and timely initiation of appropriate therapy. Although treatment completion was associated with improved survival on unadjusted analysis, it did not remain independently significant after adjustment, suggesting that treatment modality and stage at diagnosis may have a stronger influence on outcomes.

The strengths of our study include a relatively large cohort, comprehensive survival analysis and evaluation of multiple demographic, clinical and treatment-related predictors. However, certain limitations should be acknowledged. Being a retrospective hospital-based study, the findings may not be fully generalisable to the population level. Additionally, data on molecular markers, lifestyle factors and quality of life outcomes were limited. Future multicentric prospective studies incorporating molecular profiling and survivorship outcomes are required for a better understanding of the disease biology and to optimise treatment strategies in the Indian population. Several studies that were referenced are mentioned in Supplementary Table 1.

Overall, our findings emphasise the critical importance of early detection, timely initiation of multimodality treatment and strengthening cancer care infrastructure to improve colon cancer survival outcomes in India.

## Conclusion and recommendations

Colon cancer survival in this tertiary care cohort was relatively high, with a 5-year OS of 71%. Advanced stage at diagnosis and signet ring cell histology were significant predictors of poor survival, while multimodality treatment independently improved outcomes.

The study highlights the critical influence of stage at diagnosis and tumour biology on survival outcomes. It provides evidence that survival can be substantially improved in resource-limited settings when patients receive comprehensive, multidisciplinary treatment. The findings also suggest that the demographic and socioeconomic factors may indirectly influence outcomes through their impact on stage at presentation and treatment.

Strengthening early detection strategies, promoting awareness of symptoms and ensuring timely access to multidisciplinary treatment are essential to improve survival outcomes. Expanding screening initiatives and improving treatment accessibility, particularly for advanced disease, should be in the Indian healthcare setting.

## Conflicts of interest

The authors have no relevant financial or non-financial interests to disclose.

## Funding

The authors declare that no funds, grants or other support were received during the preparation of this manuscript.

## Figures and Tables

**Supplementary Table 1.** Colon cancer survival rates from published studies.

S. No	Author (Ref No.)	Year	Population	Study design	Sample size	5-year survival
1	Yeole *et al* [4]	1987–1991	India	Retrospective cohort (registry-based)	1,642	36.6%
2	Allemani *et al* [19]	2000–2014	Global	Pooled analysis of registries (retrospective)	37,513,025	~60% (varies)
3	Arnold *et al* [2]	1990–2010	Global	Systematic analysis / secondary data analysis	—	~50%–65%
4	Edwards *et al* [14]	1975–2010	USA	Retrospective cohort (registry-based)	—	~65%
5	Singh and Jemal [13]	1950–2014	USA	Secondary data analysis	—	~60%–65%
7	Hugen *et al* [8]	2000–2010	Europe	Retrospective cohort	—	~30%–40%
8	Hyngstrom *et al* [16]	1998–2010	USA	Retrospective cohort	—	~30%–50%
9	Patil *et al* [10]	2010–2015	India	Retrospective cohort	—	~40%–55%
11	Agrawal *et al* [20]	2015–2022	India	Retrospective observational study	—	~50%–60%
12	Cancer Today (IARC) [3]	2020	Global	Database/registry summary (descriptive)	—	~50%–65%

**Table 1.** Sociodemographic and clinical characteristics of study participants (N = 434).

Sociodemographic factors	Number (%)	Clinical factors	Number (%)
Age (Mean ± SD)	50 ± 13.58	Diagnostic method	
<44 years	145 (33%)	Microscopic	426 (98.0%)
45–60 years	168 (39%)	Based on Outside	7 (1.61%)

>60 years	121 (28%)	Radiologically	1 (0.23%)
Gender		Primary histology	
Male	278 (64%)	Adenocarcinoma	355 (81.7%)
Female	156 (36%)	Signet ring cell carcinoma	37 (8.5%)
Education		Mucinous carcinoma	34 (7.8%)
School and below	281 (65%)	Clinical	1 (0.2%)
College and above	152 (35%)	Others	7 (1.6%)
Occupation		Histological grade	
Employed	358 (82%)	Well differentiated	255 (59%)
Unemployed	21 (5%)	Poorly differentiated	82 (18.89%)
Retired	52 (12%)	Undifferentiated	97 (22.35%)
Income (INR)		Treatment type	
<10,000	229 (53%)	Single modality	284 (65%)
≥10,000	205 (47%)	Combined modality	150 (35%)
Religion		Treatment completion	
Hindu	341 (78.5%)	Complete treatment	367 (85%)
Muslim	68 (15.6%)	Partial treatment	67 (15%)
Christian	13 (2.99%)	Type of case	
Others	12 (2.76%)	New	263 (61%)
Region		Old	171 (39%)
North	57 (13.3%)	Clinical extent	
South	8 (2%)	Localised	18 (4.14%)
Central	37 (9%)	Regional	143 (32.94%)
East	142 (33%)	Distant mets	102 (23.50%)
Northeast	24 (6%)	Prior RX (Prior authorization)	171 (39.40%)
West	145 (33.41%)	Payment mode	
Foreign	21 (5%)	General	233(54%)
Marital status		Private	201(46%)
Married	387 (89%)		
Unmarried	25 (6%)		
Others	22 (5%)		

**Table 2.** OS based on sociodemographic and clinical characteristics.

Characteristic	Category	n	1 year (%)	3 years (%)	5 years (%)	p-value
Overall		434	88	77	71	0.04
Age (years)	<44	145	88	77	75	
	45–60	168	86	79	77	
	>60	121	88	71	58	
Gender	Male	278	88	71	71	0.82
	Female	156	87	74	71	
Clinical extent	Early stage	161	97	93	89	<0.001
	Advanced stage	102	73	50	43	
Primary histology	Adenocarcinoma	355	89	80	74	0.002
	Signet ring cell	37	68	42	42	
	Mucinous	34	87	64	64	
Grade	Well- differentiated	255	90	85	79	<0.001
	Poorly differentiated	82	86	68	66	
	Undifferentiated	97	81	53	51	
Type of treatment	Single modality	284	84	71	64	0.001
	Combined modality	150	93	85	81	
Treatment completion	Complete treatment	367	89	80	74	<0.001
	Partial treatment	67	77	50	50	
Comorbid conditions	Yes	156	83	71	63	0.02
	No	273	89	79	75	

**Table 3.** Unadjusted and adjusted hazard ratios for OS.

S. No	Variable	Category	N	Unadjusted hazard ratio (95% CI)	p-value	Adjusted hazard ratio (95% CI)	p-value
1	Age in category	<44	145	1.00		1.00	
		45–60	168	0.96 (0.59–1.58)	0.89	0.77 (0.45–1.31)	0.34
		>60	121	1.61 (1.01–2.58)	0.04	1.22 (0.72–2.07)	0.45
2	Gender	Male	278	1.00		1.00	
		Female	156	1.04 (0.70–1.56)	0.82	0.87 (0.57–1.32)	0.52
3	Education	Schooling and below	281	1.00		1.00	
		College and above	152	1.24 (0.84–1.85)	0.26	1.03 (0.68–1.55)	0.87
4	Primary histology	Adenocarcinoma	355	1.00		1.00	
		Signet ring cell	37	2.82 (1.64–4.85)	<0.001	1.95 (1.06–3.59)	0.03
		Mucinous	34	1.37 (0.71–2.65)	0.34	0.93 (0.46–1.87)	0.85
		Clinical	1	3.3 (---)	1.00	3.5 (0)	0
		Others	7	0.59 (0.83–4.30)	0.61	0.21 (0.29–1.61)	0.13
5	Grade	Well- differentiated	255	1.00		1.00	
		Poorly differentiated	82	1.9 (1.17–3.15)	<0.01	1.35 (0.78–2.36)	0.27
		Undifferentiated	97	2.9 (1.90–4.60)	<0.001	1.60 (0.94–2.71)	0.07
6	Clinical extent	Early stage	161	1.00		1.00	
		Advanced stage	102	8.73 (4.75–16.0)	<0.001	7.42 (3.67–14.9)	<0.001
7	Treatment completion	Complete	367	1.00		1.00	
		Partial	67	2.31 (1.45–3.68)	<0.001	1.60 (0.98–2.62)	0.06
8	Type of treatment	Single modality	284	1.00		1.00	
		Combined modality	150	0.41 (0.26–0.66)	<0.001	0.48 (0.25–0.91)	<0.001
9	Comorbidities	Yes	156	1.00		1.00	
		No	273	0.61 (0.42–0.90)	0.01	0.71 (0.46–1.1)	0.13
